# Are all neuroscience degrees the same? A comparison of undergraduate neuroscience degrees across the United Kingdom

**DOI:** 10.1177/23982128241307585

**Published:** 2024-12-18

**Authors:** Isabel M. Logan, Charlotte Mosley, Thomas Malcomson, Emma Yhnell

**Affiliations:** School of Biosciences, Cardiff University, Cardiff, United Kingdom

**Keywords:** Education, undergraduate, neuroscience, credit allocation, core module, optional module, accreditation

## Abstract

Considering the broad scope covered by the field of neuroscience, this study compares neuroscience undergraduate degree programmes across the United Kingdom, with a focus on the distribution of core and optional neuroscience-specific modules. Data from 13 universities were analysed; this revealed significant variation in the proportion of NS module credits acquired by graduation, ranging from 28% to 100% across institutions. The findings highlight particularly low core NS content in Year 1, potentially affecting informed choice of subsequent modules. The observed flexibility in module selection throughout a neuroscience undergraduate degree is a promising opportunity for students to explore their interdisciplinary interests. However, in response to the high variability in NS core and total credits demonstrated by this research, this study calls for further discussion on establishing an accreditation framework to ensure consistency in neuroscience undergraduate degrees across the United Kingdom.

## Introduction

It is a pivotal time for Higher Education (HE) across the United Kingdom, with HE providers facing significant financial challenges (Chapman and Dearden, 2021; Frank and Gowar, 2019). Although many HE providers are trying to attract more students to attempt to address their financial challenges, neuroscience degrees are typically expensive to offer, given the substantial cost of consumables and associated staff time to provide hands-on lab-based practical training (Jefferson, 2021; Office for Students, 2018). Furthermore, entry-level qualifications can vary across HE providers, and potential neuroscience applicants commonly have little or no experience of previously studying the subject. Therefore, establishing the relative merits, benefits and opportunities of different neuroscience courses can be complex, particularly when different phrasing and descriptions are used by HE providers.

### To accredit or not to accredit?

In the United Kingdom, some undergraduate science courses may be accredited and recognised by professional bodies. For example, biomedical sciences can be accredited by the Institute of Biomedical Sciences (IBMS) (2023). If universities and departments choose to accredit their undergraduate courses, there may be a range of benefits, including benchmarking the standard of education, supporting education and training with independent expertise, and facilitating peer recognition of best practice and dissemination through additional networks. However, the accreditation of courses will often have significant cost implications, as universities will need to pay the professional bodies for accreditation, and it may limit or restrict what can be taught to benchmark educational content. Despite accreditations being offered across some science subjects, undergraduate courses in neuroscience do not currently have accreditation in the United Kingdom.

### A comparison of undergraduate neuroscience degrees across the United Kingdom

Within the biosciences, neuroscience is a comparatively broad topic, drawing on subjects from cellular and molecular biology to cognition, computational approaches, gene-inspired technologies, genomics, neuroimaging and cross-species translation to give just a few examples; therefore, specific teaching focus and subject expertise can vary substantially across institutions. However, this breadth of content, and the lack of professional regulation or accreditation, necessitates a discussion of whether neuroscience students across the United Kingdom are being grounded in the same core knowledge and skills. Important questions remain, as to what these skills are, and how the variation in available neuroscience content to achieve a neuroscience degree may impact not only the student experience but also the ability for graduates to engage with the wider field and employment opportunities upon graduating.

## Methods

### Data search strategy

All data searches were conducted based on freely and publicly available information accessible online. The British Neuroscience Association (BNA) (n.d.-a) was used as an initial resource to consider information on all ‘Neuroscience (BSc)’ undergraduate courses offered across the United Kingdom. Information from the BNA website was used by two undergraduate researchers to subsequently search individual university websites to find more detailed and specific information for each neuroscience course. The identified courses were then cross-referenced with courses on the Universities and Colleges Admissions Service (UCAS) website to verify that no institutions were unduly omitted.

### Data collection

Publicly available data relating to ‘Neuroscience (BSc)’ courses, provided on each university website, was considered, and the following information was collected by each individual researcher on 3 June 2024:

- Course name.
- Length of course (years).
- Course modules (core and optional).
- Module description information (core and optional).
- Credits for each module (core and optional).
- Any unique course features (e.g. compulsory year in industry).

The data were compared to ensure consistency and to improve the credibility of data collection between researchers. Data were then discussed and refined with the project team to ensure accuracy, relevance and reliability of interpretation.

### Data analysis

University names were anonymised, and data were analysed according to module name, description and associated credits.

### Determining neuroscience-specific modules

Module titles and descriptions were reviewed by the research team and subsequently classified as ‘neuroscience specific (NS)’, or not, based on title and the module description. For example, modules which included words such as ‘neuroscience’, ‘nervous system’, ‘neurodegeneration’, ‘neuro-’ or ‘nervous’ in either their title or module description were classified as NS. For final year dissertations and research projects, it was assumed that these were NS based on the degree taken (unless otherwise stated in the descriptions) and characterised as core credits. Furthermore, modules which contained significant elements of psychology were also classified as NS based on substantial coverage within a neuroscience context (examples include modules relating to the neurobiology of mental illness). The NS categorisations were further checked by academic staff to provide a second opinion and to clarify and agree on any modules which were unclear.

### Establishing module credits

During data collection, it was clear that not all university websites state the specific number of credits each module is worth publicly on their websites. Therefore, courses where the specific number of credits for each module were not explicitly stated or did not equate to 120 credits per year (The Quality Assurance Agency (QAA) for Higher Education, 2021) were excluded from the analysis. Once these exclusion criteria were applied, 13 of the original 21 courses considered remained for the data analysis (Table 1).

**Table 1.** Neuroscience-specific (NS) module content in neuroscience undergraduate degree programmes across the United Kingdom.

University	Year 1		Year 2		Year 3	
	NS credits taken of total core credits	Minimum (maximum) NS credits taken of total optional credits	NS credits taken of total core credits	Minimum (maximum) NS credits taken of total optional credits	NS credits taken of total core credits	Minimum (maximum) NS credits taken of total optional credits
University A	30 of 120	None	60 of 90	0 of 30	45 of 45	0 (75)
University B	40 of 100	0 (20)	60 of 80	0 (20)*	40 of 60	40 (60)
University C	120 of 120	None	120 of 120	None	90 of 90	30
University D	0 of 80	0 (40)	20 of 60	0 (20)*	80 of 80	0 (40)
University E	30 of 120	None	60 of 90	0 (30)	45 of 45	0 (75)
University F	45 of 120	None	45 of 60	0 (45)*	None	120
University G	15 of 120	None	75 of 105	15 (15)	60 of 60	30 (60)
University H	0 of 60	0 (60)	120 of 120	None	60 of 60	0 (60)
University I	0 of 120	None	80 of 80	0 (40)	90 of 90	0*
University J	60 of 120	None	90 of 120	None	90 of 90	0*
University K	0 of 100	0 (20)	30 of 75	0 (45)	120 of 120	None
University L	45 of 120	None	60 of 60	30 (45)*	75 of 75	15 (45)
University M	30 of 120	None	105 of 120	None	60 of 60	0 (15)*
**Average**	**32**	**0 (11)**	**71**	**3 (20)**	**66**	**18 (35)**
**Std Dev**	**32.0**	**0 (18.6)**	**30.2**	**8.6 (18.4)**	**29.0**	**32.6 (28.5)**

Variation in module content between neuroscience courses across UK universities, including differences in core and optional module credit weightings. Core and optional module proportions were calculated for each year of a 3-year undergraduate degree. All years were normalised to 120 credits for both core and optional modules for each year.

NS: neuroscience specific.

* At least one non-NS module is needed to acquire 120 credits. ‘None’ indicates that no optional modules were available to be taken. Standard deviations describe the variation between the respective minimum and maximum values and do not account for possible intermittent values arising from students’ module choice.

### Additional criteria

During data analysis, a number of considerations and modifications were made due to inconsistencies noted within the data collected. For example, for Scottish universities, the first year of an undergraduate degree typically acts as a foundation year; therefore, the first year was disregarded, and Years 2–4 were used as comparators to align with the data collected from English and Welsh universities in Years 1–3. For universities with mandatory placement years, these years were excluded from the analysis, and the other 3 years excluding the placement year were considered. As such, during analysis, ‘Year 1’ refers to the highest pre-honours year, and ‘Years 2 and 3’ refer to the two taught honours years. A final check was conducted by cross-referencing the collected data with university website pages before presenting the results as shown in Table 1.

## Results

As shown in Table 1, there is significant variation in core and optional neuroscience-specific (NS) modules comprised in the different neuroscience undergraduate degrees across the United Kingdom. Furthermore, the minimum and maximum NS content taught over the course of a whole programme varied significantly, ranging from a minimum of 28% of course credits to a maximum of 100% of course credits, as seen in Table 2.

**Table 2.** Percentage of neuroscience-specific (NS) credits taken during an undergraduate neuroscience degree programme.

University	Full course		Honours years	
	Minimum NS credits at graduation (%)	Maximum NS credits at graduation (%)	Minimum NS credits at graduation (%)	Maximum NS credits at graduation (%)
University A	38	58	44	75
University B	33	67	58	75
University C	100	100	100	100
University D	28	44	42	67
University E	38	58	44	88
University F	58	71	69	88
University G	54	63	75	88
University H	50	67	75	100
University I	47	47	71	88
University J	67	67	75	75
University K	42	42	63	81
University L	63	75	75	94
University M	54	58	69	75
Average	52	63	66	84
Std Dev	17.1	12.0	15.6	10.0

Minimum and maximum values of credits possible from NS modules, as a percentage of total credits, during undergraduate neuroscience courses. Full course values are calculated as ‘(Year 1 + Year 2 + Year 3)/360’, whereas Honours years values are calculated as ‘(Year 2 + Year 3)/240’, omitting Year 1 credits. Standard deviations describe the variation between the respective minimum and maximum values and do not account for possible intermittent values arising from students’ module choice.

### Variation in core modules over the course of a neuroscience degree

Data analysis revealed that there is significant variation in core NS content across Year 1 of undergraduate degrees. For example, in Year 1, University C has 120 credits of NS core content, whereas Universities D, H, I and K have 0 credits of NS core content, compared to an average of 32 credits of NS core content across all universities included in the analysis.

The credits acquired from core NS content increase in Year 2, with no universities having 0 NS core credits. However, there remains substantial variation ranging from a minimum of 20 credits of NS core content for University D to a maximum of 120 credits of NS core content for Universities C and H. Overall, Year 2 NS core content increased with an average of 71 credits of NS core content across all universities included in the analysis.

Comparatively, Year 3 shows only a small increase in NS core content, in comparison to Year 2, with an average of 75 credits of NS core content and a small reduction in the variation between institutions (Table 1).

### Variation in optional modules over the course of a neuroscience degree

The majority of universities included in the analysis have no requirement for optional NS modules to be taken in any year of an undergraduate degree course in neuroscience. In contrast, Universities G and L stipulate a required amount of NS credits that must be acquired through optional modules (Table 1). Furthermore, of those universities with no Year 2 requirement for optional module selection, only three Universities (B, C and F) require students to take NS optional modules in Year 3, with University F being an outlier as, while all 120 Year 3 credits must be NS, all credits are from optional modules.

There is, however, a steady increase in optional NS modules across the years of undergraduate study. Nine universities offer no optional NS modules in Year 1; five universities in Year 2, where each allocates >90 credits to NS core modules; and three universities in Year 3, where, again, each allocates >90 to NS core modules (Table 1) therefore demonstrating high NS content but comparatively low optionality.

### Neuroscience module content differs between pre-honours and honours years

Table 2 highlights the effect of the relatively limited NS module content in Year 1 on the total makeup of undergraduate neuroscience degree courses when pre-honours and honours years are considered. Analysis revealed that the average minimum percentage of NS credits across honours years is 66%, when Year 1 is included, this falls to an average of just over half at 52%, while maintaining a similar level of variation across institutions. Furthermore, Universities D, H and K offer NS optional modules but do not ‘require’ NS content be taken in Year 1, whereas University I offers no NS content (core or optional) in Year 1.

## Discussion

### Significant variability in the content of neuroscience undergraduate courses across the United Kingdom

The significant variability in the content of neuroscience undergraduate courses taught across the United Kingdom, demonstrated throughout this study, may emerge due to differences in staff expertise, training and willingness to teach outside of their immediate subject areas. Furthermore, this may be exacerbated due to a lack of an accreditation framework for neuroscience in the United Kingdom. In addition, the variation in NS core credits available to students in Year 1 may result in vastly different exposures to NS content when students are beginning to decide their future optional modules, further interests and subsequent specialisations.

Furthermore, the comparable variation found in NS core credits in Years 2 and 3 (Table 1) raises the question of whether neuroscience graduates across the United Kingdom are achieving the same foundational neuroscience knowledge, skills and education, on top of any institutional specialisations. In addition, when credits from Year 1 are omitted, as Year 1 is typically not considered in the Honours degree classifications (Table 2), there are three Universities (A, D and E) where a neuroscience degree can be achieved with <50% of credits being acquired from NS modules. These findings create a strong argument for an accreditation framework to benchmark core neuroscience content that any neuroscience graduate should be expected to be able to draw upon by the time they graduate. Such an accreditation framework may also have specific, or additional, benefits for students entering commercial sectors, in a similar way to those seen in psychology where an accredited route is required for those pursuing clinical paths but not required for other careers. Furthermore, an accreditation framework may lessen the potential risk of students being limited in their options for future or postgraduate study based on the expertise or focus of their institution by guaranteeing a consistent grounding in neuroscience subject knowledge.

### Variability in the teaching content may not necessarily be a bad thing

Our analysis demonstrates that, broadly, variation in neuroscience degrees across the United Kingdom can be placed in three categories:

1. Those requiring a set amount of core NS credits but allowing students flexibility to integrate non-NS optional modules (the majority; e.g. Universities J and M).
2. Those requiring NS core credits and stipulating that a set number of optional credits be acquired from only NS optional modules (e.g. Universities G and L).
3. Those with no requirement for optional NS credits across the course (e.g. Universities A, D, E and H).

However, variability in NS content across an undergraduate course may not necessarily be a bad thing. Undergraduate degrees with more optionality often receive positive feedback from students on their flexibility, and they may allow and enable students to explore additional content outside of neuroscience to supplement their own interest in the field. As such, while it is unlikely that courses containing 0% mandated NS content would be ideal, there is significant room for discussion as to what would be considered an optimal range of NS content to enable an appropriate grounding in the neuroscience field, while also allowing students flexibility and autonomy over their areas of interest.

Given both the broad nature of neuroscience as a field, and the diverse interests and careers of neuroscience graduates British Neuroscience Association (BNA) (n.d.-b), the capacity to tailor the subject focus of their modules may be powerful in aiding students to develop their knowledge and skills that can be applied to their futures. However, given the high degree of variation in NS content observed in undergraduate neuroscience degrees across the United Kingdom, the need for an evaluation of what constitutes foundational knowledge and skills, and whether these are being covered consistently, is clearly warranted. Finally, any accreditation framework would need to account not only for foundational knowledge and skills but also acknowledge the quality of educational delivery and experience for undergraduate students.
